# Patterns of Infarction on MRI in Patients With Acute Ischemic Stroke and Cardio-Embolism: A Systematic Review and Meta-Analysis

**DOI:** 10.3389/fneur.2020.606521

**Published:** 2020-12-08

**Authors:** Angelos Sharobeam, Leonid Churilov, Mark Parsons, Geoffrey A. Donnan, Stephen M. Davis, Bernard Yan

**Affiliations:** ^1^Melbourne Brain Centre at the Royal Melbourne Hospital, Parkville, VIC, Australia; ^2^Faculty of Medicine, Dentistry and Health Sciences, The University of Melbourne, Parkville, VIC, Australia; ^3^Florey Institute of Neuroscience and Mental Health, The University of Melbourne, Parkville, VIC, Australia; ^4^South Western Sydney Clinical School, The University of New South Wales, Liverpool, NSW, Australia; ^5^Department of Neurology, Liverpool Hospital, Liverpool, NSW, Australia; ^6^The Ingham Institute, Liverpool, NSW, Australia

**Keywords:** stroke, cardio-embolism, magnetic resonance imaging, topography, volume

## Abstract

**Background:** Cardioembolic strokes are common however atrial fibrillation, the most common cause, is often asymptomatic and difficult to detect. There is evidence that infarct topography and volume on magnetic resonance imaging may be associated with specific stroke etiologies.

**Aim:** A systematic review and meta-analysis were undertaken to summarize the available evidence on the association between stroke etiology, infarct topography, and volume.

**Methods:** A systematic review was conducted using Medline (OVID), Embase (OVID), and PubMed databases. Hand searches of the gray literature and of reference lists in relevant articles were also performed. A quality assessment was undertaken, based on the STROBE checklist. For each study, the number of patients with and without a CE source of stroke and infarct topography was collected and outcomes presented as odds ratios (OR) with 95% CI and *p*-values.

**Results:** Four thousand eight hundred and seventy-three patients with ischemic stroke were included, of whom 1,559 were determined to have a CE source. Bilateral infarcts (OR 3.41; 95% CI 2.20–5.29; *p* < 0.0001) and multiple territory infarcts (OR 1.57; 95% CI 1.12–2.21; *p* = 0.009) were more common in patients with a CE source of stroke, than patients without a CE source. Lacunar infarcts (OR 0.49; 95% CI 0.31–0.80; *p* = 0.004) were more likely to occur in patients without a CE source. No significant difference between the frequency of multiple infarcts (OR 0.96; 95% CI 0.57–1.61; *p* = 0.87) anterior circulation (OR 1.45; 95% CI 0.83–2.53; *p* = 0.19) or posterior circulation infarcts (OR 1.06; 95% CI 0.72–1.57; *p* = 0.75), between the two groups were identified. Three out of four studies examining volume, found a significant association between increased infarct volume and CE source of stroke. A sensitivity analysis with cryptogenic and undetermined stroke sources assumed to be cardioembolic, did not alter the associations observed.

**Conclusion:** The findings of this systematic review and meta-analysis are broadly consistent with previous literature and provide more robust evidence on the association between infarct topography, volume and stroke etiology. Our findings may assist with refining cardiac investigations for patients with cryptogenic stroke, based on infarct topography.

## Introduction

Ischemic stroke is a common cause of morbidity globally, with over 116 million years of healthy life lost each year due to stroke related death and disability (1). Cardioembolic (CE) sources form the underlying etiology in 20–25% of cases (2). Atrial fibrillation (AF), a common cause of CE stroke, may be asymptomatic in one third of patients and hence may go undetected (3). This has major implications for management, given the proven benefit of anticoagulation (4, 5). Current diagnostic techniques for AF detection are limited by low detection rate, particularly with non-invasive techniques such as Holter monitoring and wearable devices (6). Cost effectiveness if also a factor. The test with the highest detection rate, an implantable cardiac monitor (ICM), is not cost-effective with the incremental cost-effectiveness ratio (ICER) at £17,175 per quality of life year (QALY) gained, compared to 24-h cardiac telemetry (7).

Previous studies have examined factors, which predict the presence of AF in patients with ischemic stroke. Older age, atrial cardiopathy (8), and a prolonged PR interval on ECG, are independently associated with increased incidence of AF (9–11). Serum biomarkers, such as brain natriuretic peptide, have been associated with future development of paroxysmal AF in patients with ischemic stroke (12, 13). Thrombus histopathology has also been associated with stroke etiology, with CE source strokes more likely to be fibrin rich and erythrocyte poor (14, 15). In addition to findings from cardiac and pathology testing, there is evidence to suggest that a CE stroke etiology may be associated with topographical infarct patterns on magnetic resonance imaging (MRI).

Multi-territory MRI-DWI lesion topography is a common finding in CE strokes (16–18). In a large study by Chung et al. (17), multi-territory infarction was more prevalent amongst those with a CE etiology (representing 44.2% of all multi-territory infarctions). A number of other studies have demonstrated that a large proportion of patients with multi-territory infarction will have a CE source (19, 20). Akhtar et al. (19), in a systematic review of simultaneous infarcts in multiple territories, revealed that simultaneous infarcts in the anterior and posterior circulation, are more likely to be associated with AF than other etiologies such as large artery atherosclerosis (LAA). Such infarcts had an underlying CE source in two-thirds of cases.

A study by Yushan et al. (16) using MRI–DWI imaging demonstrated that 30% of patients with detected AF had bilateral infarcts at presentation, significantly higher than the 5.5% of the control cohort. Studies looking at anterior circulation infarcts did not show a clear predilection for CE stroke as opposed to other stroke etiologies (21–23). A study by Rizos et al., utilizing lesion mapping techniques, showed a higher probability of CE infarcts in the right insula compared to other regions (24).

A number of studies (25–27) examining infarct patterns in the posterior circulation revealed LAA was the most likely etiology, however CE etiologies were implicated in 20–30% of cases. In contrast, Chung et al. (17) showed that 60% of superior cerebellar artery infarcts have an underlying CE source. Schiphorst et al. (28), however, showed that small obliquely oriented cortical cerebellar infarcts have a higher risk of future diagnosis of AF, compared to other cerebellar infarct subsets.

Larger infarct volumes have been associated with CE source of stroke, in multiple studies (24, 29–31). In addition to infarct size, subsequent infarct growth, and hemorrhagic transformation are also strongly predictive of underlying CE source. In a secondary analysis of the EPITHET trial by Tu et al. (29), patients with definite AF had significantly greater infarct growth, larger infarcts, more frequent parenchymal hematoma grade hemorrhagic transformation, worse functional outcomes and higher mortality compared to patients with no AF. An analysis of infarct volume by Rizos et al. (24) likewise showed larger volumes in patients with both new and established AF.

### Hypothesis/Study Question

In patients with acute ischemic stroke, infarct pattern and volume on MRI will differ between patients with and without a CE source.

## Methods

### Literature Search

**Figure 1:**
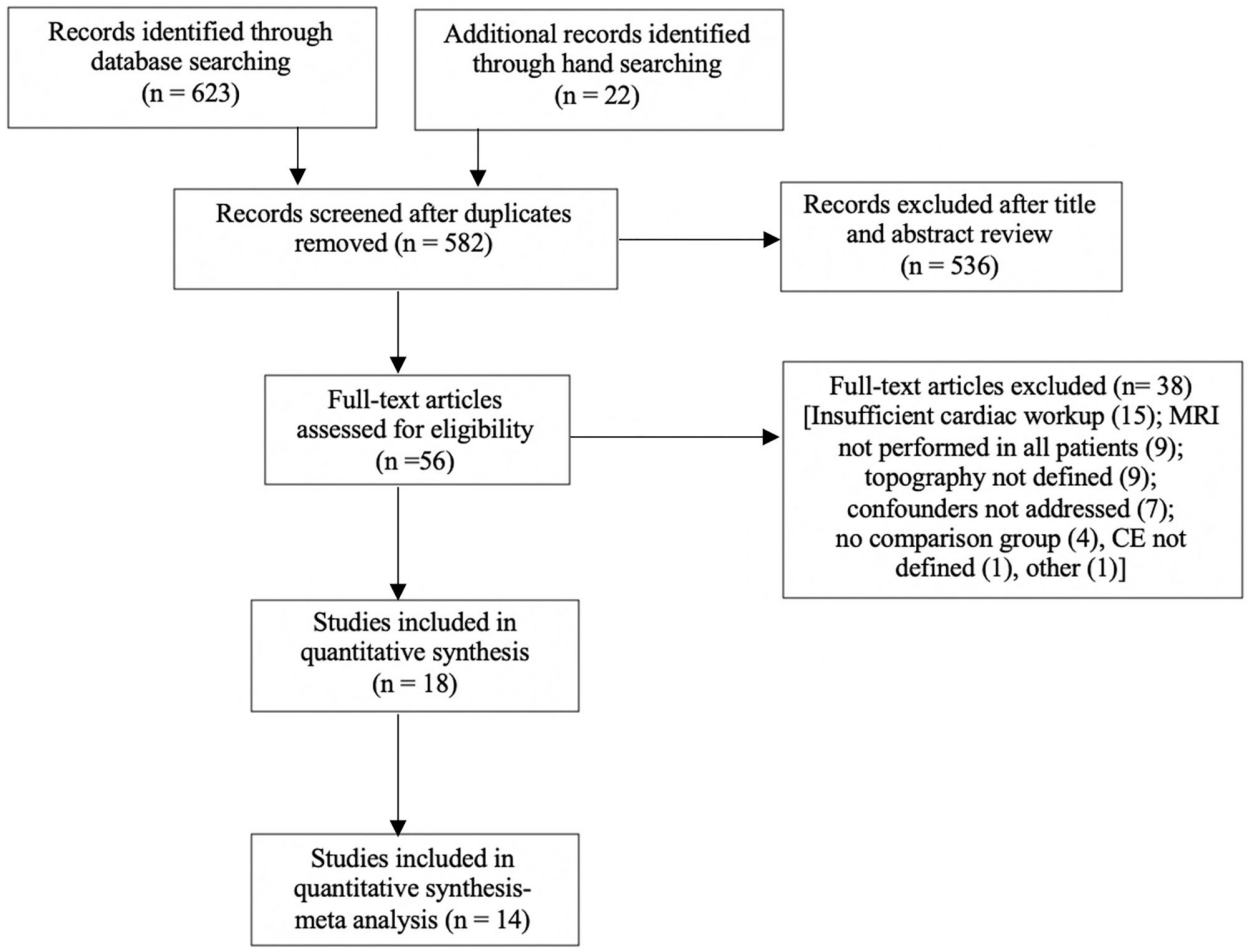
Search strategy for meta-analysis. Full text articles analyzed, may have more than one reason for exclusion. Eighteen articles were included, with four excluded from the meta-analysis due to analysis of volume only.

A systematic review was performed according to PRISMA guidelines (32) and registered with the PROSPERO register of systematic reviews (Registration ID CRD42020169479). The search strategy (Supplementary Material 1) included a combination of controlled synonyms and vocabularies from Medical Subject Headings (MeSH) and EmTree covering stroke, infarction, pattern, topography, distribution, atrial fibrillation, cardioembolism, magnetic resonance imaging, and diffusion weighted imaging. The search was conducted using Medline (OVID), Embase (OVID), and PubMed databases using a combination of truncations and Boolean operators. Hand searches of the gray literature and of reference lists in relevant articles were also performed. Two co-authors reviewed the abstracts and selected the included studies (AS, BY). The literature search excluded reviews, editorials, and conference abstracts. The search strategy is summarized in Figure 1.

### Inclusion Criteria

A quality assessment of all full text articles selected was undertaken (Supplementary Table 1 in Supplementary Material 2) based on The Strengthening the Reporting of Observational Studies in Epidemiology (STROBE) checklist and Joanna Briggs Institute checklists for cross-sectional studies (33, 34). To be included in the meta-analysis, all studies were required to meet the following minimum criteria:

1. All patients had an MRI with diffusion weighted imaging (DWI) sequence performed.
2. Infarct topography is clearly defined.
3. The criteria for a stroke categorized as cardio-embolic is clearly defined. For example, using the TOAST criteria (35).
4. All included patients have been investigated for cardio-embolic sources, at a minimum with baseline ECG and 24 h of cardiac telemetry plus echocardiography if no cause found.
5. A comparison group is available, i.e., cardioembolic vs. non-cardioembolic strokes.
6. Background and objectives are clearly delineated.
7. Participant selection criteria are outlined.
8. Appropriate synthesis, outcome measures, methodology, and statistics.
9. Confounders and limitations are addressed.

### Data Extraction

For each study, the total number of patients with CE stroke, the total number of stroke patients, the breakdown of stroke etiologies and topographical infarct patterns on MRI, were collected. Stroke etiology was dichotomized as being either CE or non-CE source. Where etiology was undetermined, the cause was assumed to be non-CE. A sensitivity analysis with the opposite assumption, with undetermined etiology assumed to be CE, was therefore performed which revealed no difference in the results (Supplementary Material 3). Topographical patterns analyzed were bilateral infarcts, multiple infarcts, multiple vascular territory infarcts, lacunar, anterior circulation, posterior circulation, internal watershed, and external watershed infarcts. Papers mentioning infarct volume, were also analyzed.

### Statistical Analysis

Review Manager (RevMan) version 5.3 (Copenhagen: The Nordic Cochrane Center, The Cochrane Collaboration, 2014) was used to perform a meta-analysis of observational studies fulfilling the above criteria. The event rate for individual topographical patterns and 95% confidence intervals (CI) were determined from each study. Each topographical pattern was analyzed separately. Outcomes are presented as odds ratios (OR) with 95% CI and *p*-values. We used the Mantel-Haenszel implementation of the DerSimonian and Laird random-effect method implemented in RevMan (36). This implementation estimates the amount of between-study variation by comparing each study's result with a Mantel-Haenszel fixed-effect meta-analysis result (36). Heterogeneity of treatment effect across studies was evaluated by using the *I*^2^ statistic, in which *I*^2^ > 50% suggests substantial heterogeneity, as per the guide for interpretation of thresholds for *I*^2^ from the Cochrane handbook chapter 10 (36). A *p* < 0.05 was treated as indicative of statistical significance. Funnel plot asymmetry and Egger's test was used to illustrate the extent of small study publication bias (Supplementary Material 4).

## Results

The literature search yielded 632 articles. Following review of titles, abstracts, exclusion of conference abstracts, and review articles, 45 full text papers were considered for inclusion. Fourteen papers exploring topography fulfilled all inclusion criteria (16, 18, 26, 37–47) and were included in a meta-analysis.

**Figure 2:**
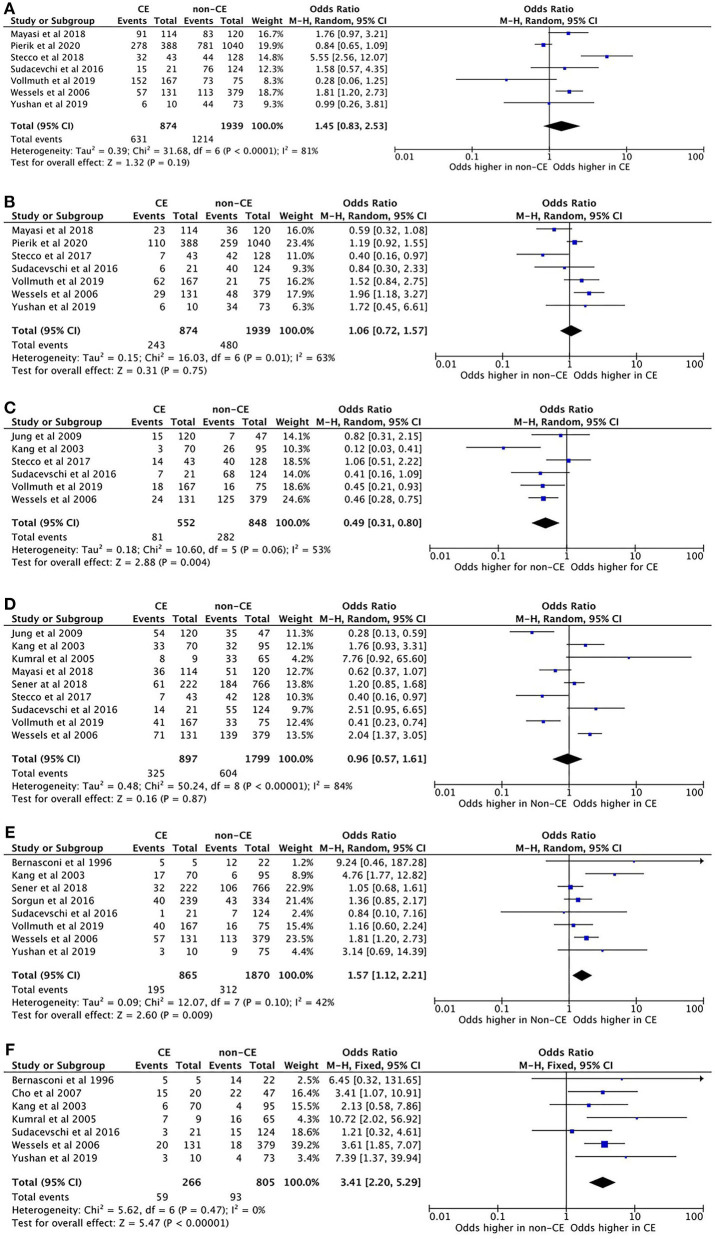
**(A)** Forest plot showing the odds ratio of anterior circulation infarct topography in CE stroke, compared to non-CE stroke. **(B)** Forest plot showing the odds ratio of posterior circulation infarct topography in CE stroke, compared to non-CE stroke. **(C)** Forest plot showing the odds ratio of lacunar infarct topography in CE stroke, compared to non-CE stroke. **(D)** Forest plot showing the odds ratio of multiple infarcts in one or more arterial territories in CE stroke, compared to non-CE stroke. **(E)** Forest plot showing the odds ratio of infarcts in multiple large artery territories in CE stroke, compared to non-CE stroke. **(F)** Forest plot showing the odds ratio of bilateral hemisphere infarcts in CE stroke, compared to non-CE stroke.

A total of 4,873 patients with ischemic stroke were included, of whom 1,559 were determined to have a CE source. Bilateral infarcts (OR 3.41; 95% CI 2.20–5.29; *p* < 0.0001) and multiple territory infarcts (OR 1.57; 95% CI 1.12–2.21; *p* = 0.009) were more common in patients with a CE source of stroke, than patients without a CE source. Lacunar infarcts (OR 0.49; 95% CI 0.31–0.80; *p* = 0.004) were more likely to occur in patients without a CE source. No significant difference between the frequency of multiple infarcts (OR 0.96; 95% CI 0.57–1.61; *p* = 0.87) anterior circulation (OR 1.45; 95% CI 0.83–2.53; *p* = 0.19) or posterior circulation infarcts (OR 1.06; 95% CI 0.72–1.57; *p* = 0.75), between the two groups were identified (Figures 2A–F). No significant heterogeneity was detected in the bilateral and multiple territory subgroups; however, the other subgroups did display significant heterogeneity between the included studies. Egger's test did not reveal any evidence of publication bias. Only one paper (39) analyzed watershed infarcts. Of 43 patients with watershed infarcts, 27 were classified as external watershed and 16 as internal watershed. In both cases, a non-CE etiology was more common than a CE etiology. Volume was not included in the meta-analysis, due to the low number of studies included and non-uniform reporting methods.

A sub-analysis of multiple territory and bilateral infarcts was performed on papers which also reported chronic infarcts in these territories. Only one paper reported on the presence of concurrent chronic infarcts (44). There were numerically more chronic multi-territory infarcts seen in the non-AF population (15/83, 18%) than the AF population (2/21, 9.5%), although this did not reach statistical significance.

### Infarct Volume

Four papers meeting inclusion criteria also analyzed infarct volume (2, 4, 29–31). The median baseline volume in CE ranged from 8.3 ml in Kim et al. (30) to 22.5 ml in Tu et al. (29) and the non-CE ranged from 3.0 ml in Kim et al. (30) to 4.2 ml in Tu et al. (29). In two out of three studies (29, 30), the baseline infarct volume with statistically significantly higher in CE compared to non-CE stroke. Rizos et al. (24) reported a mean baseline infarct volume of 41.2 ml, which was likewise significantly higher than patients with non-CE source of stroke (15.1 ml).

## Discussion

We set out to determine differences between infarct pattern and volume. in patients with and without a CE source of stroke. The findings of our study indicate that larger infarct volumes and certain topographical patterns, including multiple territory and bilateral infarcts in any arterial territory, occur more commonly in patients with a CE source of stroke. Lacunar infarcts are more likely to be associated with a non-CE source. These findings support previous studies in which a correlation between a CE source and infarcts in multiple territories, including bilateral infarcts (16–20) was found. This association to CE stroke may be explained by atrial thrombus fragmentation, leading to infarcts in different arterial territories (46).

Although the causes of multi-territory infarcts do include non-CE etiologies (44), the association of non-CE etiologies with other patterns in our meta-analysis, particularly lacunar, would support the notion that non-CE source infarct topography is less likely to be multi-territory compared to CE source strokes.

The lack of association with either a CE or non-CE etiology in anterior circulation and posterior circulation strokes, may be due to the influence of multiple vascular risk factors, such as diabetes and ischemic heart disease influencing infarct location (48). One question that remains unanswered however is whether or not a CE source, such as AF, is more likely to determine topography, when two or more etiologies are considered equally likely. The results of our sensitivity analysis, which showed no difference in patterns, would suggest that intercurrent CE sources offer no additional weighting to the topography of the infarct.

Studies analyzing volume, all indicated that larger infarct volumes are more likely to be associated with a cardio-embolic source. This finding supports the findings of Tu (49), who postulated that larger infarct volumes were the result of poorer collateral circulation in patients with AF.

Strengths of our study include the definitions of topography and volume based on MRI alone, rather than CT, and the inclusion of a large number of patients (4,873), which reduces the likelihood that topographical associations were due to chance alone. The comparison to other possible stroke sources and the criteria for minimum cardiac investigations, reduces the likelihood of a missed CE source.

An urgent need exists for rapid, non-invasive, accurate tests for AF detection. There is an unanswered question regarding anticoagulation for cryptogenic stroke, particularly those with embolic stroke of undetermined source (ESUS). The findings of our study are pertinent given negative studies for anticoagulation is ESUS so far (50, 51). The results of our analysis suggest the cryptogenic source strokes offer no additional weighting to infarct source based on MRI topography. In line with recent literature (52, 53), this may in turn indicate that a large portion of cryptogenic strokes may actually be non-cardioembolic and therefore will not benefit from anticoagulation.

## Limitations

There were a number of limitations to this study. First and foremost, in all studies, the etiology of ischemic stroke could not be determined with absolute certainty, as this would necessitate screening for all potential stroke risk factors in all patients. Although PRISMA guidelines were adhered to, there is a risk of selection and reporting bias, as all studies are observational and the majority retrospective in nature. Not all included studies, reported on all topographical patterns, hence comparison was made on available data. Infarct patterns were often common to multiple groups; for example, patients with multi-territory infarcts also had anterior or posterior circulation infarcts recorded. It was often not possible to determine the proportion of isolated infarcts in different circulations, as this was often not explicitly stated (with the exception of lacunar infarcts). Other confounders, such as anticoagulant and statin use, could not be accounted for. There was significant heterogeneity in a number of subgroups, likely reflecting the inclusion of studies with low patient numbers.

## Conclusions and Future directions

This study has provided further evidence for associations between infarct volume, topography and CE stroke. Further large, long term prospective studies, with low cardiovascular risk populations, are needed to demonstrate this link. The use of more sensitive methods for determining infarct topography and volume than human visual analysis, such as machine learning, may also improve our understanding of infarct etiology in future. The use of machine learning in stroke neuroimaging is already well-established, being utilized for determination of ischemic penumbra and large vessel occlusions (54). Emerging applications include the prediction of stroke onset time, functional outcomes following stroke, neurological deterioration, and hemorrhagic transformation (55). Future studies utilizing machine learning in infarct topography may offer additional, more accurate diagnostic tools for determination of stroke etiology. This may in turn lead to future anticoagulation trials in patients with high risk cardioembolic patterns, looking for stroke recurrence.

In the interim, this study may assist with refining cardiac investigations for patients with cryptogenic stroke, based on infarct topography.

## Data Availability Statement

The original contributions presented in the study are included in the article/Supplementary Materials, further inquiries can be directed to the corresponding author.

## Author Contributions

AS conceptualized the project and was responsible for primary writing of the text. LC and AS performed the statistical analysis and co-wrote the article methods. AS and BY selected and critically appraised included articles. SD, GD, LC, MP, and BY provided critical appraisal and editing of the manuscript. All authors approved the final version of the manuscript.

## Conflict of Interest

The authors declare that the research was conducted in the absence of any commercial or financial relationships that could be construed as a potential conflict of interest. The handling Editor declared a past co-authorship with one of the authors LC.

## References

[1] Johnson CO Nguyen M Roth GA Nichols E Alam T Abate D Global, regional, and national burden of stroke, 1990–2016: a systematic analysis for the Global Burden of Disease Study 2016. Lancet Neurol. (2019) 18:439–58. 10.1016/S1474-4422(19)30034-1 30871944 PMC6494974

[2] O'Donnell MJ Chin SL Rangarajan S Xavier D Liu L Zhang H Risk factors for ischemic and intracerebral haemorrhagic stroke in 22 countries (the INTERSTROKE study): a case-control study. Lancet. (2010) 376:112–23. 10.1016/S0140-6736(10)60834-3 20561675

[3] Dilaveris PE Kennedy HL. Silent atrial fibrillation: epidemiology, diagnosis and clinical impact. Clin Cardiol. (2017) 40:413–8. 10.1002/clc.22667 28273368 PMC6490532

[4] Hart RG Pearce LA Aguilar MI. Meta-analysis: antithrombotic therapy to prevent stroke in patients who have nonvalvular atrial fibrillation. Ann Intern Med. (2007) 146:857–67. 10.7326/0003-4819-146-12-200706190-00007 17577005

[5] van Walraven C Hart RG Singer DE Laupacis A Connolly S Petersen P. Oral anticoagulants vs aspirin in nonvalvular atrial fibrillation: an individual patient meta-analysis. JAMA. (2002) 288:2441–8. 10.1001/jama.288.19.2441 12435257

[6] Schnabel RB Haeusler KG Healey JS Freedman B Boriani G Brachmann J Searching for atrial fibrillation poststroke: a white paper of the AF-SCREEN International Collaboration. Circulation. (2019) 140:1834–50. 10.1161/CIRCULATIONAHA.119.040267 31765261

[7] Diamantopoulos A Sawyer LM Lip GY Witte KK Reynolds MR Fauchier L. Cost-effectiveness of an insertable cardiac monitor to detect atrial fibrillation in patients with cryptogenic stroke. Int J Stroke. (2016) 11:302–12. 10.1177/1747493015620803 26763916

[8] Kamel H Bartz TM Elkind MS Okin PM Thacker EL Patton KK. Atrial cardiopathy and the risk of ischemic stroke in the CHS (Cardiovascular Health Study). Stroke. (2018) 49:980–6. 10.1161/STROKEAHA.117.020059 29535268 PMC5973804

[9] Thijs VN Brachmann J Morillo CA Passman RS Sanna T Bernstein RA. Predictors for atrial fibrillation detection after cryptogenic stroke: results from crystal AF. Neurology. (2016) 86:261–9. 10.1212/WNL.0000000000002282 26683642 PMC4733152

[10] Poli S Diedler J Hartig F Gotz N Bauer A Sachse T. Insertable cardiac monitors after cryptogenic stroke–a risk factor based approach to enhance the detection rate for paroxysmal atrial fibrillation. Eur J Neurol. (2016) 23:375–81. 10.1111/ene.12843 26470854

[11] Haeusler KG Groschel K Kohrmann M Anker SD Brachmann J Bohm M. Expert opinion paper on atrial fibrillation detection after ischemic stroke. Clin Res Cardiol. (2018) 107:871–80. 10.1007/s00392-018-1256-9 29704214

[12] Scheitz JF Erdur H Haeusler KG Audebert HJ Roser M Laufs U. Insular cortex lesions, cardiac troponin, and detection of previously unknown atrial fibrillation in acute ischemic stroke: insights from the troponin elevation in acute ischemic stroke study. Stroke. (2015) 46:1196–201. 10.1161/STROKEAHA.115.008681 25835563

[13] Rodríguez-Yáñez M Arias-Rivas S Santamaría-Cadavid M Sobrino T Castillo J Blanco M. High pro-BNP levels predict the occurrence of atrial fibrillation after cryptogenic stroke Neurology. (2013) 81:444–7. 10.1212/WNL.0b013e31829d8773 23803318

[14] Sporns PB Hanning U Schwindt W Velasco A Minnerup J Zoubi T. Ischemic stroke: what does the histological composition tell us about the origin of the thrombus? Stroke. (2017) 48:2206–10. 10.1161/STROKEAHA.117.016590 28626055

[15] Maekawa K Shibata M Nakajima H Mizutani A Kitano Y Seguchi M. Erythrocyte-rich thrombus is associated with reduced number of maneuvers and procedure time in patients with acute ischemic stroke undergoing mechanical thrombectomy. Cerebrovasc Dis Extra. (2018) 8:39–49. 10.1159/000486042 29402828 PMC5836222

[16] Yushan B Tan BYQ Ngiam NJ Chan BPL Luen TH Sharma VK. Association between bilateral infarcts pattern and detection of occult atrial fibrillation in Embolic Stroke of Undetermined Source (ESUS) in patients with Insertable Cardiac Monitor (ICM). J Stroke Cerebrovasc Dis. (2019) 28:2448–52. 10.1016/j.jstrokecerebrovasdis.2019.06.025 31307898

[17] Chung JW Hyun S Kim N Kim WJ Park JH Ko Y. Trial of ORG 10172 in Acute Stroke Treatment (TOAST) classification and vascular territory of ischemic stroke lesions diagnosed by diffusion-weighted imaging. J Am Heart Assoc. (2014) 3:e001119. 10.1161/JAHA.114.001119 25112556 PMC4310410

[18] Mayasi Y Helenius J McManus DD Goddeau RP Jr Jun-O'Connell AH Moonis M. Atrial fibrillation is associated with anterior predominant white matter lesions in patients presenting with embolic stroke. J Neurol Neurosurg Psychiatry. (2018) 89:6–13. 10.1136/jnnp-2016-315457 28554961 PMC5704976

[19] Akhtar T Shahjouei S Zand R. Etiologies of simultaneous cerebral infarcts in multiple arterial territories: a simple literature-based pooled analysis. Neurol India. (2019) 67:692–5. 10.4103/0028-3886.263244 31347536

[20] Depuydt S Sarov M Vandendries C Guedj T Cauquil C Assayag P. Significance of acute multiple infarcts in multiple cerebral circulations on initial diffusion weighted imaging in stroke patients. J Neurol Sci. (2014) 337:151–5. 10.1016/j.jns.2013.11.039 24332593

[21] Kang SY Kim JS. Anterior cerebral artery infarction: stroke mechanism and clinical-imaging study in 100 patients. Neurology. (2008) 70:2386–93. 10.1212/01.wnl.0000314686.94007.d0 18541871

[22] Bogousslavsky J Regli F. Anterior cerebral artery territory infarction in the lausanne stroke registry. clinical and etiologic patterns. Arch Neurol. (1990) 47:144–50. 10.1001/archneur.1990.00530020040012 2302085

[23] Sato S Toyoda K Matsuoka H Okatsu H Kasuya J Takada T. Isolated anterior cerebral artery territory infarction: dissection as an etiological mechanism. Cerebrovasc Dis. (2010) 29:170–7. 10.1159/000262314 19955742

[24] Rizos T Bartsch AJ Johnson TD Dittgen F Nichols TE Malzahn U Voxelwise distribution of acute ischemic stroke lesions in patients with newly diagnosed atrial fibrillation: trigger of arrhythmia or only target of embolism? PLoS ONE. (2017) 12:e0177474 10.1371/journal.pone.0177474 28542605 PMC5443524

[25] Amarenco P Levy C Cohen A Touboul PJ Roullet E Bousser MG. Causes and mechanisms of territorial and nonterritorial cerebellar infarcts in 115 consecutive patients. Stroke. (1994) 25:105–12. 10.1161/01.STR.25.1.105 8266355

[26] Kumral E Kisabay A Atac C Calli C Yunten N. Spectrum of the posterior inferior cerebellar artery territory infarcts. clinical-diffusion-weighted imaging correlates. Cerebrovasc Dis. (2005) 20:370–80. 10.1159/000088667 16205055

[27] Kumral E Kisabay A Ataç C. Lesion patterns and etiology of ischemia in superior cerebellar artery territory infarcts. Cerebrovasc Dis. (2005) 19:283–90. 10.1159/000084496 15775708

[28] Schiphorst AT Tatu L Thijs V Demattei C Thouvenot E Renard D. Small obliquely oriented cortical cerebellar infarctions are associated with cardioembolic stroke. BMC Neurol. (2019) 19:100. 10.1186/s12883-019-1328-0 31103038 PMC6525367

[29] Tu HT Campbell BC Christensen S Desmond PM De Silva DA Parsons MW. Worse stroke outcome in atrial fibrillation is explained by more severe hypoperfusion, infarct growth, and hemorrhagic transformation. Int J Stroke. (2015) 10:534–40. 10.1111/ijs.12007 23489996 PMC3688700

[30] Kim YD Hong HJ Cha MJ Nam CM Nam HS Heo JH. Determinants of infarction patterns in cardioembolic stroke. Eur Neurol. (2011) 66:145–50. 10.1159/000330563 21876359

[31] Jung JM Kwon SU Lee JH Kang DW. Difference in infarct volume and patterns between cardioembolism and internal carotid artery disease: focus on the degree of cardioembolic risk and carotid stenosis. Cerebrovasc Dis. (2010) 29:490–6. 10.1159/000297965 20299789

[32] Moher D Liberati A Tetzlaff J Altman DG The PRISMA Group Preferred Reporting Items for Systematic Reviews and Meta-Analyses: the PRISMA statement. PLoS Med. (2009) 6:e1000097 10.1371/journal.pmed.1000097 19621072 PMC2707599

[33] von Elm E Altman DG Egger M Pocock SJ Gotzsche PC Vandenbroucke JP The Strengthening the Reporting of Observational Studies in Epidemiology (STROBE) statement: guidelines for reporting observational studies. Ann Intern Med. (2007) 147:573–77. 10.7326/0003-4819-147-8-200710160-00010 17938396

[34] Moola S Munn Z Tufanaru C Aromataris E Sears K Sfetcu R Chapter 7: Systematic reviews of etiology and risk. In: Aromataris E Munn Z, editors. JBI Manual for Evidence Synthesis. JBI (2020).

[35] Adams HP Jr Bendixen BH Kappelle LJ Biller J Love BB Gordon DL. Classification of subtype of acute ischemic stroke. definitions for use in a multicenter clinical trial. TOAST. Trial of Org 10172 in Acute Stroke Treatment. Stroke. (1993) 24:35–41. 10.1161/01.STR.24.1.35 7678184

[36] Deeks JJ Higgins JPT Altman DG Chapter 10: analysing data and undertaking meta-analyses. In: Higgins JPT Thomas J Chandler J Cumpston M Li T Page MJ Welch VA editors. Cochrane Handbook for Systematic Reviews of Interventions Version 6.0. Cochrane (2019).

[37] Bernasconi A Bogousslavsky J Bassetti C Regli F. Multiple acute infarcts in the posterior circulation. J Neurol Neurosurg Psychiatry. (1996) 60:289–96. 10.1136/jnnp.60.3.289 8609506 PMC1073852

[38] Cho AH Kim JS Jeon SB Kwon SU Lee DH Kang DW. Mechanism of multiple infarcts in multiple cerebral circulations on diffusion-weighted imaging. J Neurol. (2007) 254:924–30. 10.1007/s00415-006-0397-3 17401747

[39] Kang DW Chalela JA Ezzeddine MA Warach S. Association of ischemic lesion patterns on early diffusion-weighted imaging with TOAST stroke subtypes. Arch Neurol. (2003) 60:1730–4. 10.1001/archneur.60.12.1730 14676047

[40] Sener U Ocek L Ilgezdi I Sahin H Ozcelik M Zorlu Y. Significance of multiple acute ischemic lesions on initial diffusion-weighted imaging in stroke patients and relation of toast classification. Ann Indian Acad Neurol. (2018) 21:197–202. 10.4103/aian.AIAN_487_17 30258262 PMC6137625

[41] Sorgun MH Togay CI Tezcan S Yilmaz V Etiologic subtypes of acute multiple infarcts in more than one vascular territories. J Neurol Sci- Turk. (2016) 33:38–44.

[42] Stecco A Quagliozzi M Soligo E Naldi A Cassara A Coppo L. Can neuroimaging differentiate PFO and AF-related cardioembolic stroke from the other embolic sources? Clinical-radiological correlation on a retrospective study. Radiol Med. (2017) 122:412–8. 10.1007/s11547-017-0738-6 28224399

[43] Sudacevschi V Bertrand C Chadenat ML Tarnaud C Pico F. Predictors of occult atrial fibrillation in one hundred seventy-one patients with cryptogenic transient ischemic attack and minor stroke. J Stroke Cerebrovasc Dis. (2016) 25:2673–7. 10.1016/j.jstrokecerebrovasdis.2016.07.014 27495831

[44] Vollmuth C Stoesser S Neugebauer H Hansel A Dreyhaupt J Ludolph AC MR-imaging pattern is not a predictor of occult atrial fibrillation in patients with cryptogenic stroke. J Neurol. (2019) 266:3058–64. 10.1007/s00415-019-09524-5 31511980 PMC6851041

[45] Wessels T Wessels C Ellsiepen A Reuter I Trittmacher S Stolz E. Contribution of diffusion-weighted imaging in determination of stroke etiology. AJNR Am J Neuroradiol. (2006) 27:35–9. 16418352 PMC7976056

[46] Pierik R Algra A van Dijk E Erasmus ME van Gelder IC Koudstaal PJ. Distribution of cardioembolic stroke: a cohort study. Cerebrovasc Dis. (2020) 49:97–104. 10.1159/000505616 31962331

[47] Maier I Schregel K Karch A Weber-Krueger M Mikolajczyk R Stahrenberg R. Association between embolic stroke patterns, ESUS etiology, and new diagnosis of atrial fibrillation: a secondary data analysis of the find-AF trial. Stroke Res Treatment. (2017) 2017:1391843. 10.1155/2017/1391843 28536667 PMC5425845

[48] Zeng Q Tao W Lei C Dong W Liu M. Etiology and risk factors of posterior circulation infarction compared with anterior circulation infarction. J Stroke Cerebrovasc Dis. (2015) 24:1614–20. 10.1016/j.jstrokecerebrovasdis.2015.03.033 25899158

[49] Tu H Stroke and atrial fibrillation: better detection, effects on infarct evolution and outcome (Ph.D. dissertation). Melbourne, VIC, Australia (2018).

[50] Diener HC Sacco RL Easton JD Granger CB Bernstein RA Uchiyama S. Dabigatran for prevention of stroke after embolic stroke of undetermined source. N Engl J Med. (2019) 380:1906–17. 10.1056/NEJMoa1813959 31091372

[51] Kasner SE Swaminathan B Lavados P Sharma M Muir K Veltkamp R. Rivaroxaban or aspirin for patent foramen ovale and embolic stroke of undetermined source: a prespecified subgroup analysis from the NAVIGATE ESUS trial. Lancet Neurol. (2018) 17:1053–60. 10.1016/S1474-4422(18)30319-3 30274772 PMC6662613

[52] Fuentes B Gutirrez-Ziga R Dez-Tejedor E. It's time to say goodbye to the ESUS construct. Front Neurol. (2020) 11:653. 10.3389/fneur.2020.00653 32733368 PMC7358305

[53] Ntaios G. Embolic stroke of undetermined source: JACC review topic of the week. J Am Coll Cardiol. (2020) 75:333–40. 10.1016/j.jacc.2019.11.024 31976872

[54] Kamal H Lopez V Sheth SA. Machine learning in acute ischemic stroke neuroimaging. Front Neurol. (2018) 9:945. 10.3389/fneur.2018.00945 30467491 PMC6236025

[55] Mouridsen K Thurner P Zaharchuk G. Artificial intelligence applications in stroke. Stroke. (2020) 51:2573–9. 10.1161/STROKEAHA.119.027479 32693750

