# Supplementary material for: Patterns of Infarction on MRI in Patients With Acute Ischemic Stroke and Cardio-Embolism: A Systematic Review and Meta-Analysis
Source: Front Neurol. 2020 Dec 8;11:606521. doi: 10.3389/fneur.2020.606521 (PMC7753023; doi:10.3389/fneur.2020.606521)
Supplement: Supplementary file 1 [file Table_1.DOCX]

Supplementary Material

1. **Search strings for systematic review**

**Ovid MEDLINE(R)**

1. exp Brain Ischemia/
2. exp Stroke/
3. (brain adj2 ischaem$).mp.
4. (brain adj2 ischem$).
5. (ischem$ adj2 stroke$).mp.
6. (ischaem$ adj2 stroke$).mp.
7. 1 or 2 or 3 or 4 or 5 or 6
8. exp Atrial Fibrillation/ or cardioembol$/ or cardio-embol$/ or card$/ or AF.mp.
9. (pattern or location or distribution or topography or volume).mp.
10. exp Magnetic Resonance Imaging/ or MRI.mp.
11. exp Diffusion Magnetic Resonance Imaging/ or DWI.mp.
12. 10 or 11
13. 7 and 8 and 9 and 12

**Database: Embase Classic + Embase**

1. exp Brain Ischemia/
2. exp Stroke/
3. (brain adj2 ischaem$).mp.
4. (brain adj2 ischem$).
5. (ischem$ adj2 stroke$).mp.
6. (ischaem$ adj2 stroke$).mp.
7. 1 or 2 or 3 or 4 or 5 or 6
8. exp Atrial Fibrillation/ or cardioembol$/ or cardio-embol$/ or card$/or AF.mp.
9. (pattern or location or distribution or topography or volume).mp.
10. exp Magnetic Resonance Imaging/ or MRI.mp.
11. exp Diffusion Magnetic Resonance Imaging/ or DWI.mp.
12. 10 or 11
13. 7 and 8 and 9 and 12

**Pubmed**

1. ((((((((((stroke) OR (infarct)) OR (infarction)) OR (ischaemia)) OR (ischaemic)) OR (ischemia)) OR (ischemia)) OR (brain ischaemia)) OR (brain ischemia)) OR (ischemic stroke)) OR (ischaemic stroke)
2. (((pattern) OR (distribution)) OR (location)) OR (topography) OR (volume)
3. (((((MRI) OR (DWI)) OR (magnetic resonance imaging)) OR (diffusion imaging) OR (diffusion weighted imaging) OR (diffusion weighted magnetic resonance imaging)
4. (((((((AF) OR (atrial fibrillation)) OR (cardioembolism)) OR (cardioembolic)) OR (cardio-embolism)) OR (cardio-embolic)) OR (cardiac source) OR (cardiac)
5. **Supplementary table- study quality**

|  | **Author** | **Year** | **Background, objectives clearly delineated?** | **Participant selection criteria outlined?** | **CE stroke defined?** | **Minimum cardiac workup complete?** | **MRI performed in all patients?** | **Topography and/or volume adequately defined?** | **Comparison group?** | **Methodology and synthesis appropriate?** | **Adjustment for potential confounders?** | **Other reasons for exclusion** |
| --- | --- | --- | --- | --- | --- | --- | --- | --- | --- | --- | --- | --- |
| 1 | Tu et al | 2015 | Y | Y | Y | Y | Y | Y | Y | Y | Y |  |
| 2 | Rizos et al | 2017 | Y | Y | Y | Y | Y | Y | Y | Y | Y |  |
| 3 | Kim et al | 2008 | Y | Y | Y | Y | Y | Y | Y | Y | Y |  |
| 4 | Jung et al | 2010 | Y | Y | Y | Y | Y | Y | Y | Y | Y |  |
| 5 | Bernasconi et al | 1996 | Y | Y | Y | Y | Y | Y | Y | Y | Y |  |
| 6 | Cho et al | 2007 | Y | Y | Y | Y | Y | Y | Y | Y | Y |  |
| 7 | Pierik et al | 2020 | Y | Y | Y | Y | Y | Y | Y | Y | Y |  |
| 8 | Kang et al | 2003 | Y | Y | Y | Y | Y | Y | Y | Y | Y |  |
| 9 | Kumral et al | 2005 | Y | Y | Y | Y | Y | Y | Y | Y | Y |  |
| 10 | Mayasi et al | 2018 | Y | Y | Y | Y | Y | Y | Y | Y | Y |  |
| 11 | Sener et al | 2018 | Y | Y | Y | Y | Y | Y | Y | Y | Y |  |
| 12 | Sorgun et al | 2016 | Y | Y | Y | Y | Y | Y | Y | Y | Y |  |
| 13 | Stecco et al | 2017 | Y | Y | Y | Y | Y | Y | Y | Y | Y |  |
| 14 | Sudacevschi et al | 2016 | Y | Y | Y | Y | Y | Y | Y | Y | Y |  |
| 15 | Vollmuth et al | 2019 | Y | Y | Y | Y | Y | Y | Y | Y | Y |  |
| 16 | Wessels et al | 2006 | Y | Y | Y | Y | Y | Y | Y | Y | Y |  |
| 17 | Yushan et al | 2019 | Y | Y | Y | Y | Y | Y | Y | Y | Y |  |
| 18 | Baird et al | 2000 | Y | Y | U | U | Y | Y | Y | Y | N |  |
| 19 | Chung et al | 2014 | Y | Y | Y | N | Y | Y | Y | Y | N |  |
| 20 | Frontzek et al | 2014 | Y | Y | Y | Y | Y | Y | Y | Y | N |  |
| 21 | Park et al | 2013 | Y | Y | Y | Y | Y | Y | Y | Y | N |  |
| 22 | Zeng et al | 2015 | Y | Y | Y | N | Y | Y | Y | Y | N |  |
| 23 | Depuydt et al | 2014 | Y | Y | Y | N | Y | Y | Y | Y | Y |  |
| 24 | Lee et al | 2000 | Y | Y | Y | N | Y | Y | Y | Y | Y |  |
| 25 | Lorance et al | 2014 | Y | Y | Y | N | Y | Y | Y | Y | N |  |
| 26 | Chowdury et al | 2004 | Y | Y | Y | N | Y | Y | Y | Y | N |  |
| 27 | Bonati et al | 2005 | Y | Y | Y | N | Y | Y | Y | Y | Y |  |
| 28 | Caso et al | 2005 | Y | Y | Y | N | Y | Y | Y | Y | Y |  |
| 29 | Kang et al | 2008 | Y | Y | Y | N | Y | Y | Y | Y | Y |  |
| 30 | Wen et al | 2004 | Y | Y | Y | N | Y | Y | Y | Y | Y |  |
| 31 | Roh et al | 2000 | Y | Y | Y | N | Y | Y | Y | Y | Y |  |
| 32 | Del bene et al | 2013 | Y | Y | Y | N | Y | Y | Y | Y | Y |  |
| 33 | Arauz et al | 2003 | Y | Y | N | N | Y | Y | Y | Y | Y |  |
| 34 | Fujimoto et al | 2011 | Y | Y | Y | Y | Y | Y | N | Y | Y |  |
| 35 | Park et al | 2014 | Y | Y | Y | Y | Y | Y | N | Y | Y |  |
| 36 | Kim et al | 2011 | Y | Y | Y | Y | Y | Y | N | Y | Y |  |
| 37 | Yasuda et al | 2018 | Y | Y | Y | Y | Y | Y | N | Y | Y |  |
| 38 | Amerenco et al | 1994 | Y | Y | Y | Y | N | Y | Y | Y | Y |  |
| 39 | Arboix et al | 2011 | Y | Y | Y | Y | N | Y | Y | Y | Y |  |
| 40 | Bernstein et al | 2015 | Y | Y | Y | Y | N | Y | Y | Y | Y |  |
| 41 | Bogousslavsky et al | 1988 | Y | Y | Y | Y | N | Y | Y | Y | Y |  |
| 42 | Cokar et al | 2007 | Y | Y | Y | Y | N | Y | Y | Y | Y |  |
| 43 | Jung et al | 2001 | Y | Y | Y | Y | N | Y | Y | Y | Y |  |
| 44 | Maier et al | 2017 | Y | Y | Y | Y | N | Y | Y | Y | Y |  |
| 45 | Miyamoto et al | 2010 | Y | Y | Y | U | N | Y | Y | Y | Y |  |
| 46 | Moulin et al | 2000 | Y | Y | Y | N | N | Y | Y | Y | Y |  |
| 47 | Eppinger et al | 2019 | Y | Y | Y | Y | Y | N | Y | Y | Y |  |
| 48 | Herm et al | 2019 | Y | Y | Y | Y | Y | N | Y | Y | Y |  |
| 49 | Kobayashi et al | 2014 | Y | Y | Y | Y | Y | N | Y | Y | Y |  |
| 50 | Yamamoto et al | 2015 | Y | Y | Y | Y | Y | N | Y | Y | Y |  |
| 51 | Schiphorst et al | 2019 | Y | Y | Y | N | Y | N | Y | Y | Y |  |
| 52 | Kass-hout et al | 2018 | Y | Y | Y | Y | Y | N | Y | Y | Y |  |
| 53 | Kumral et al | 2005 | Y | Y | Y | Y | Y | N | Y | Y | Y |  |
| 54 | Oh et al | 2011 | Y | Y | Y | Y | Y | N | Y | Y | Y |  |
| 55 | Saito et al | 2005 | Y | Y | Y | Y | Y | N | Y | Y | Y |  |
| 56 | Tu et al | 2010 | Y | Y | Y | Y | Y | Y | Y | Y | Y | Same database used in Tu et al 2015 (included) |

**Supplementary Table 1.** Full text articles analyzed with quality criteria addressed.

## 3. Supplementary Figures- Sensitivity analysis


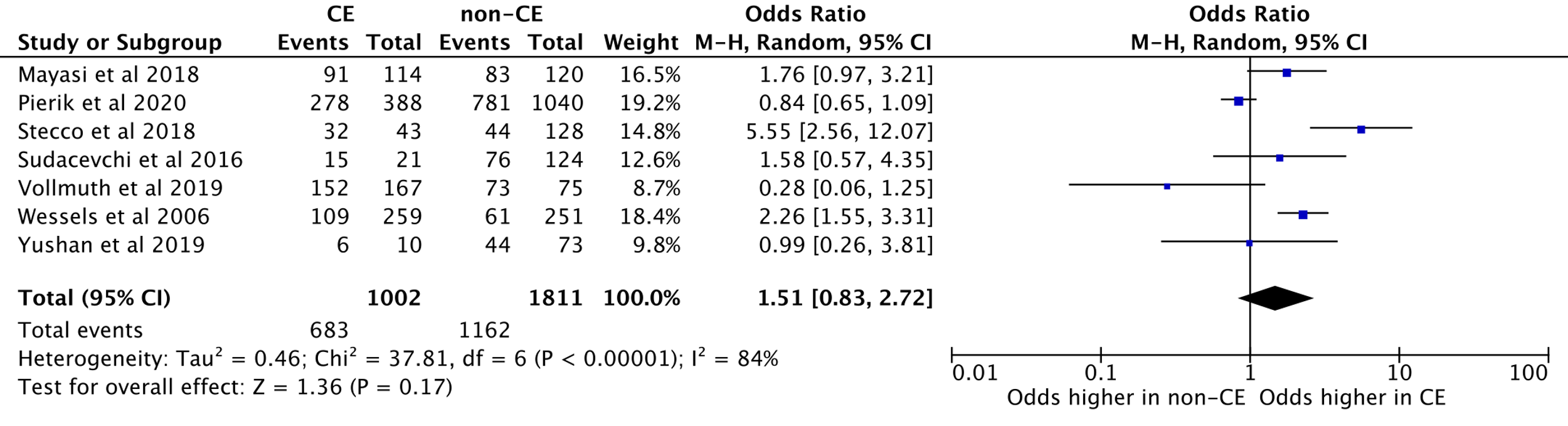


**Supplementary Figure 3(A):** Anterior circulation infarcts forest plot, with undetermined and cryptogenic strokes assumed to be CE in etiology


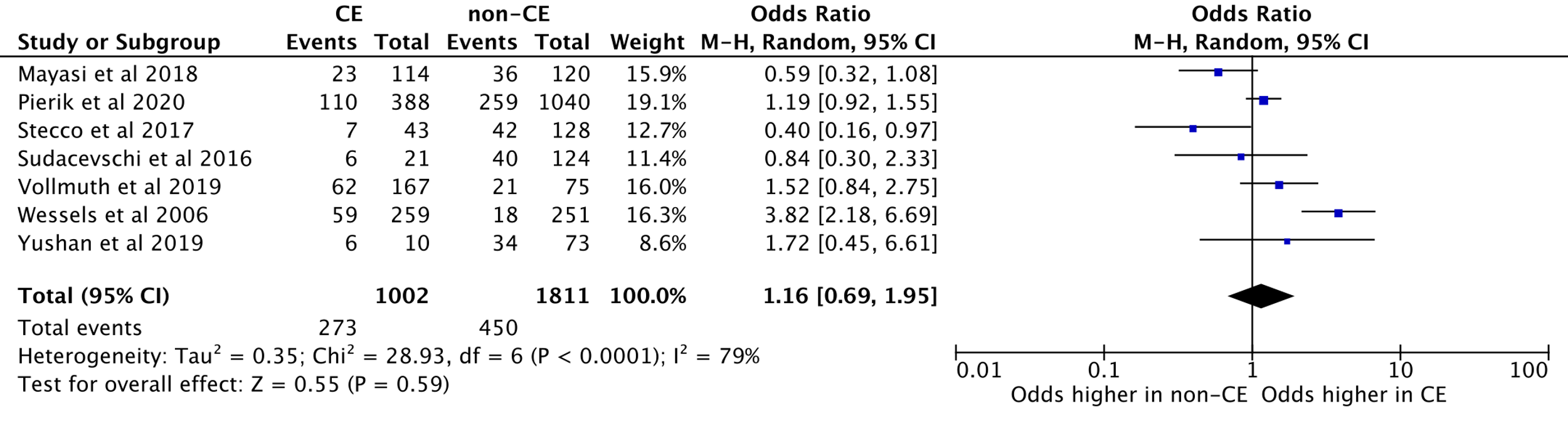


**Supplementary Figure 3(C).** Posterior circulation infarcts forest plot, with undetermined and cryptogenic strokes assumed to be CE in etiology


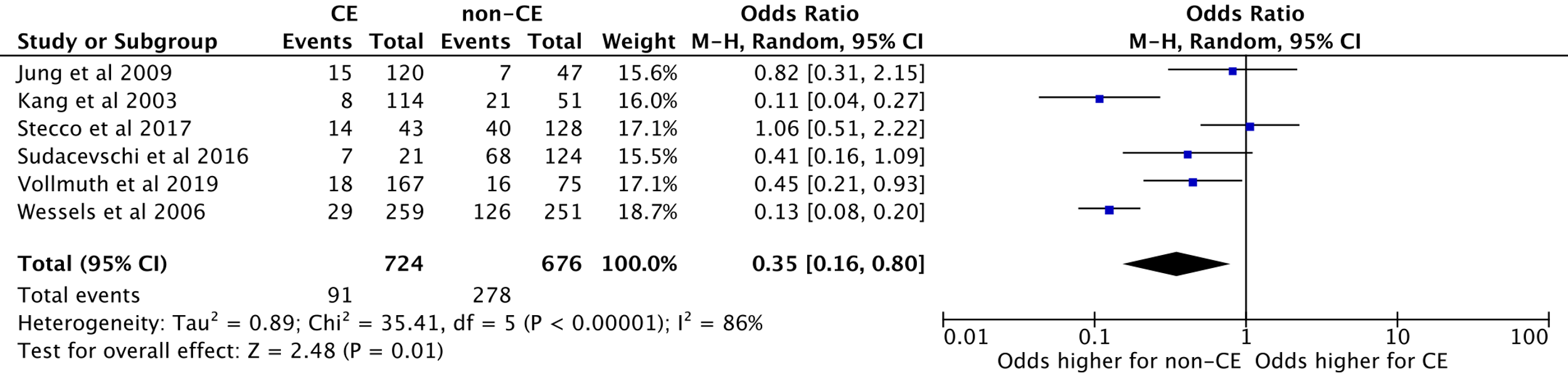


**Supplementary Figure 3(B).** Lacunar infarcts forest plot, with undetermined and cryptogenic strokes assumed to be CE in etiology


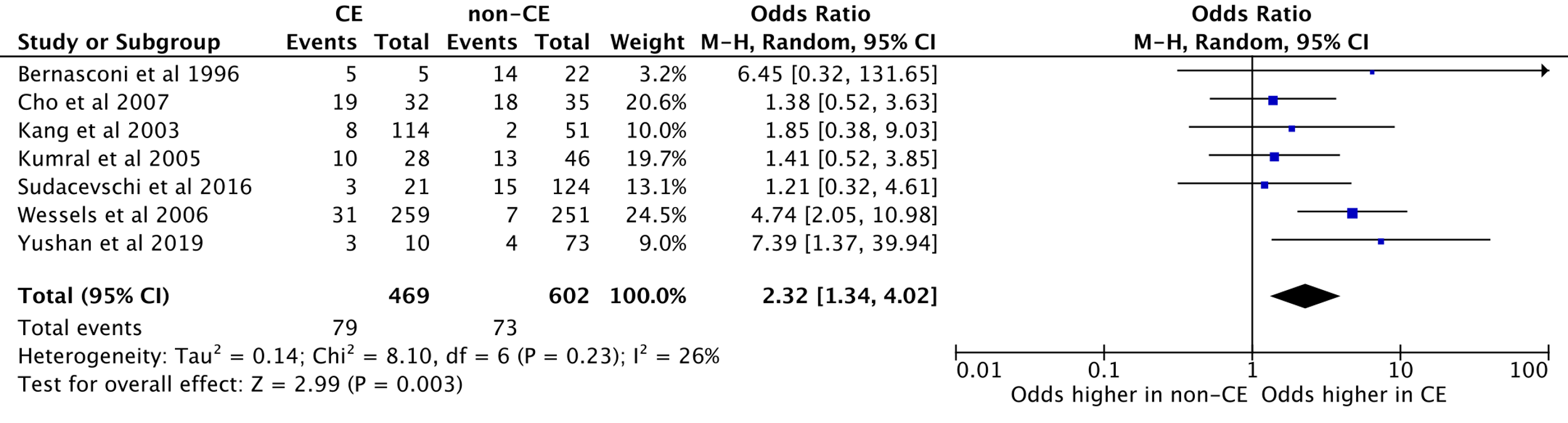


**Supplementary Figure 3(D).** Bilateral infarcts forest plot, with undetermined and cryptogenic strokes assumed to be CE in etiology


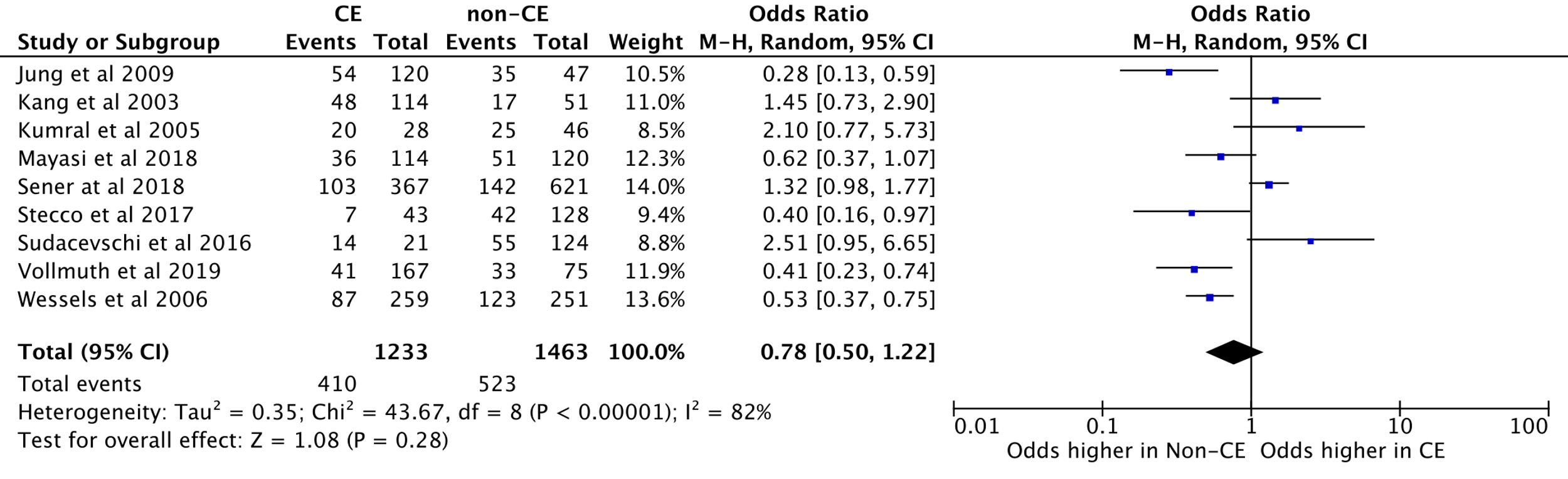


**Supplementary Figure 3(E).** Multiple infarcts forest plot, with undetermined and cryptogenic strokes assumed to be CE in etiology


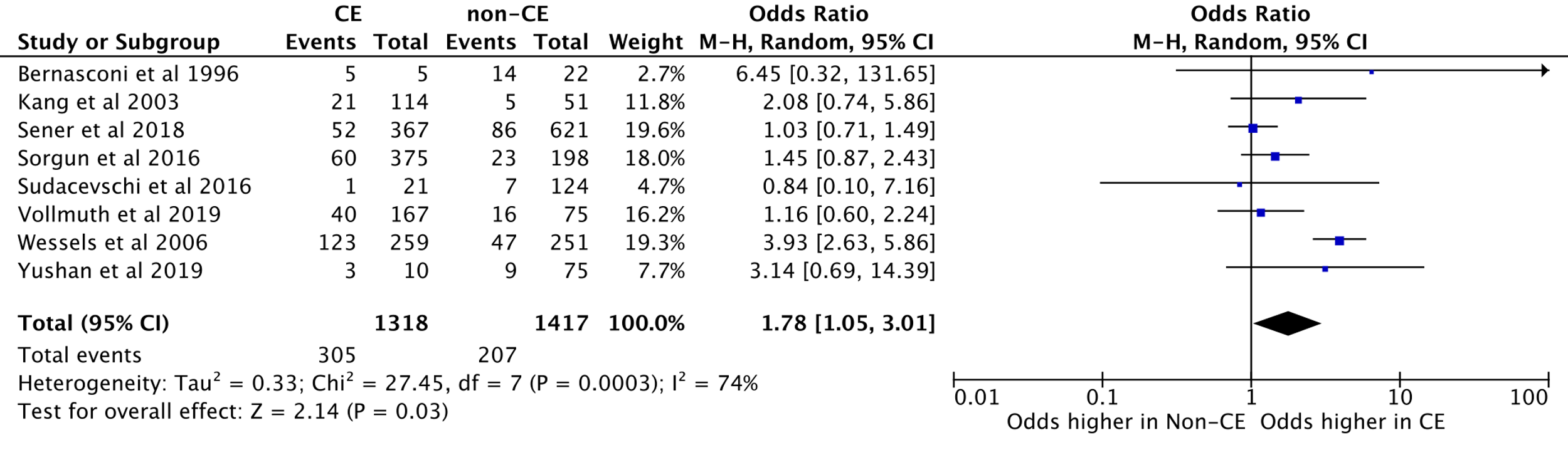


**Supplementary Figure 3(F).** Multiple territory infarcts forest plot, with undetermined and cryptogenic strokes assumed to be CE in etiology

**4- Supplementary Figures: Funnel plots**


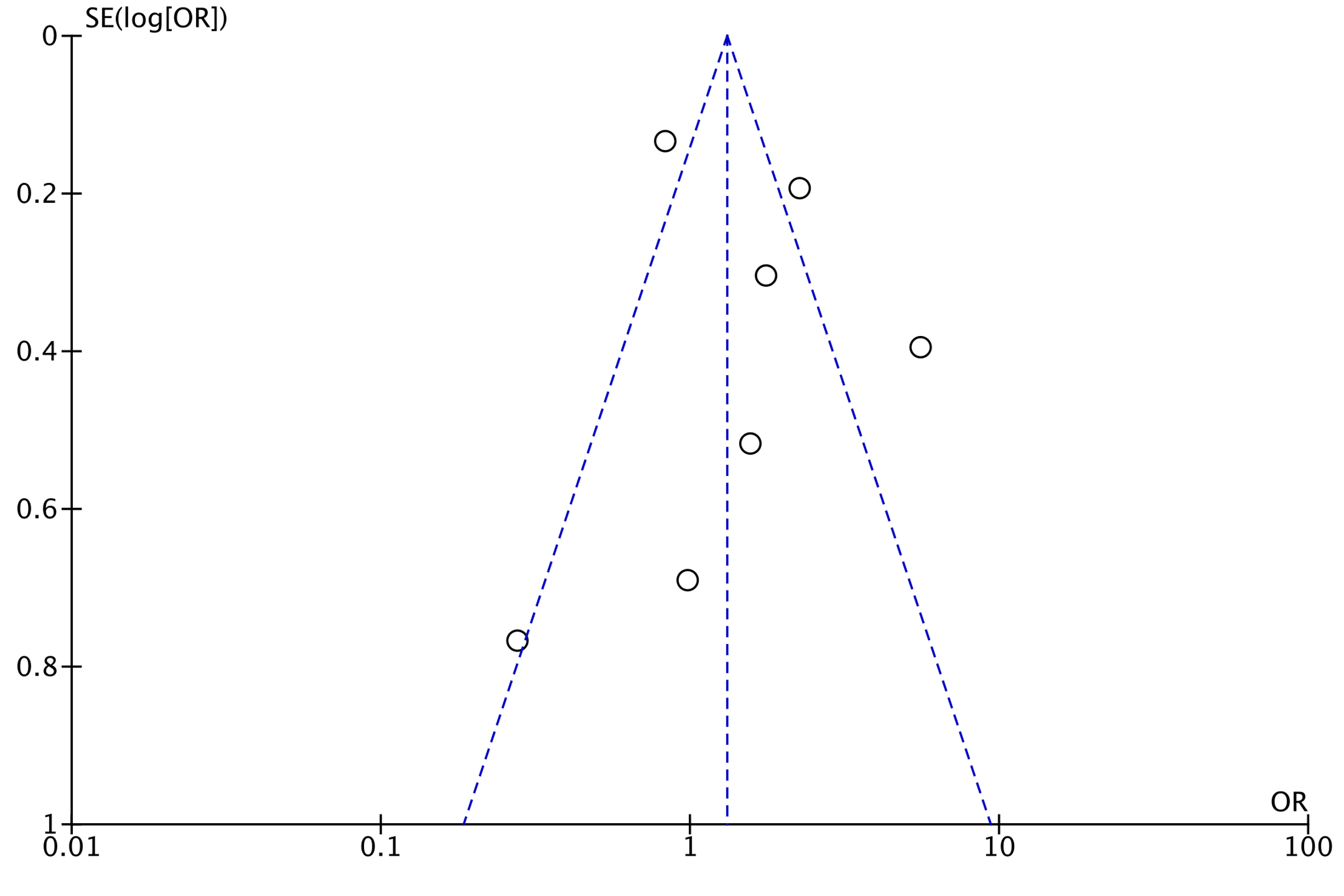


**Supplementary Figure 4(A).** Funnel plot of studies including anterior circulation infarcts


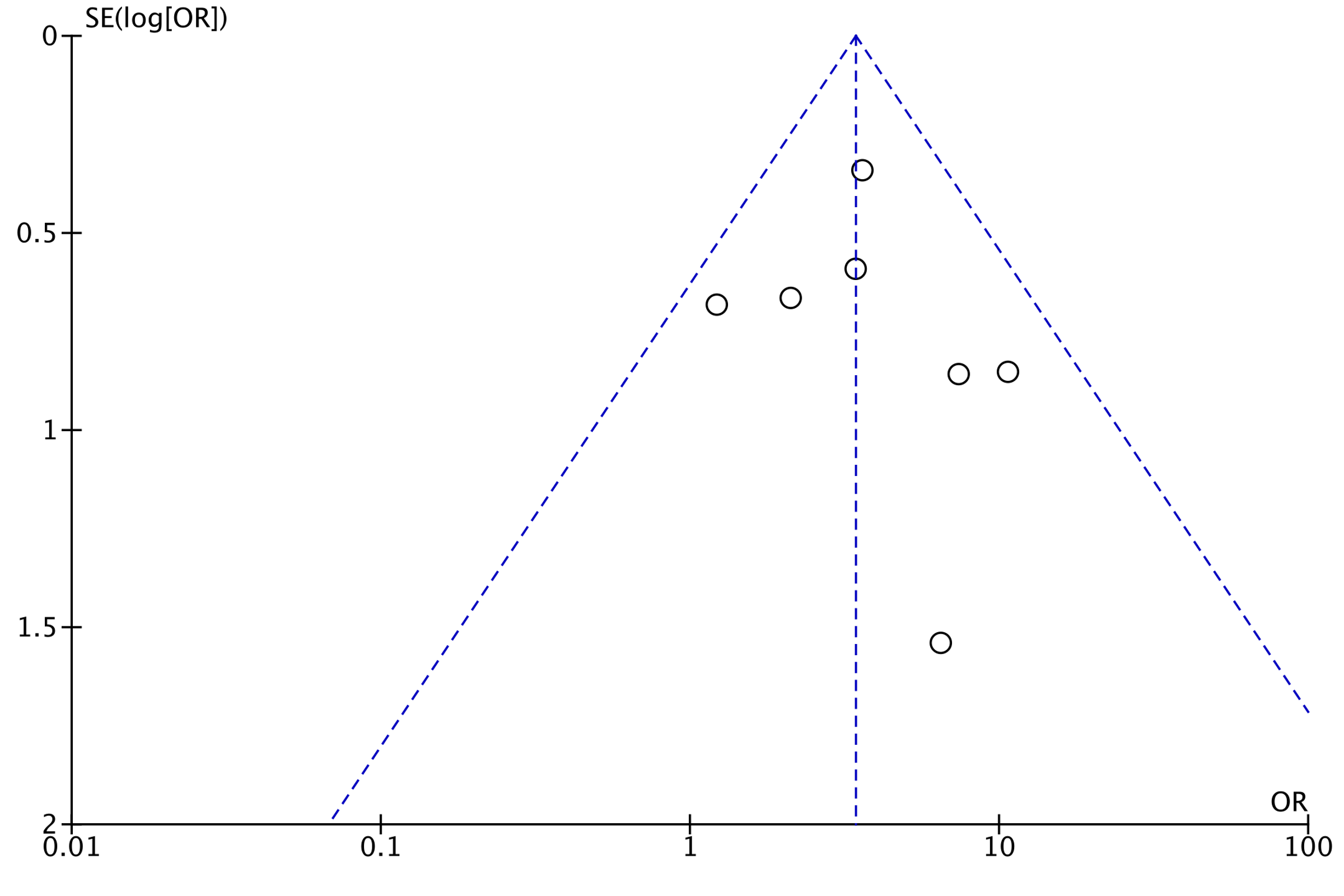


**Supplementary Figure 4(B).** Funnel plot of studies including bilateral infarcts


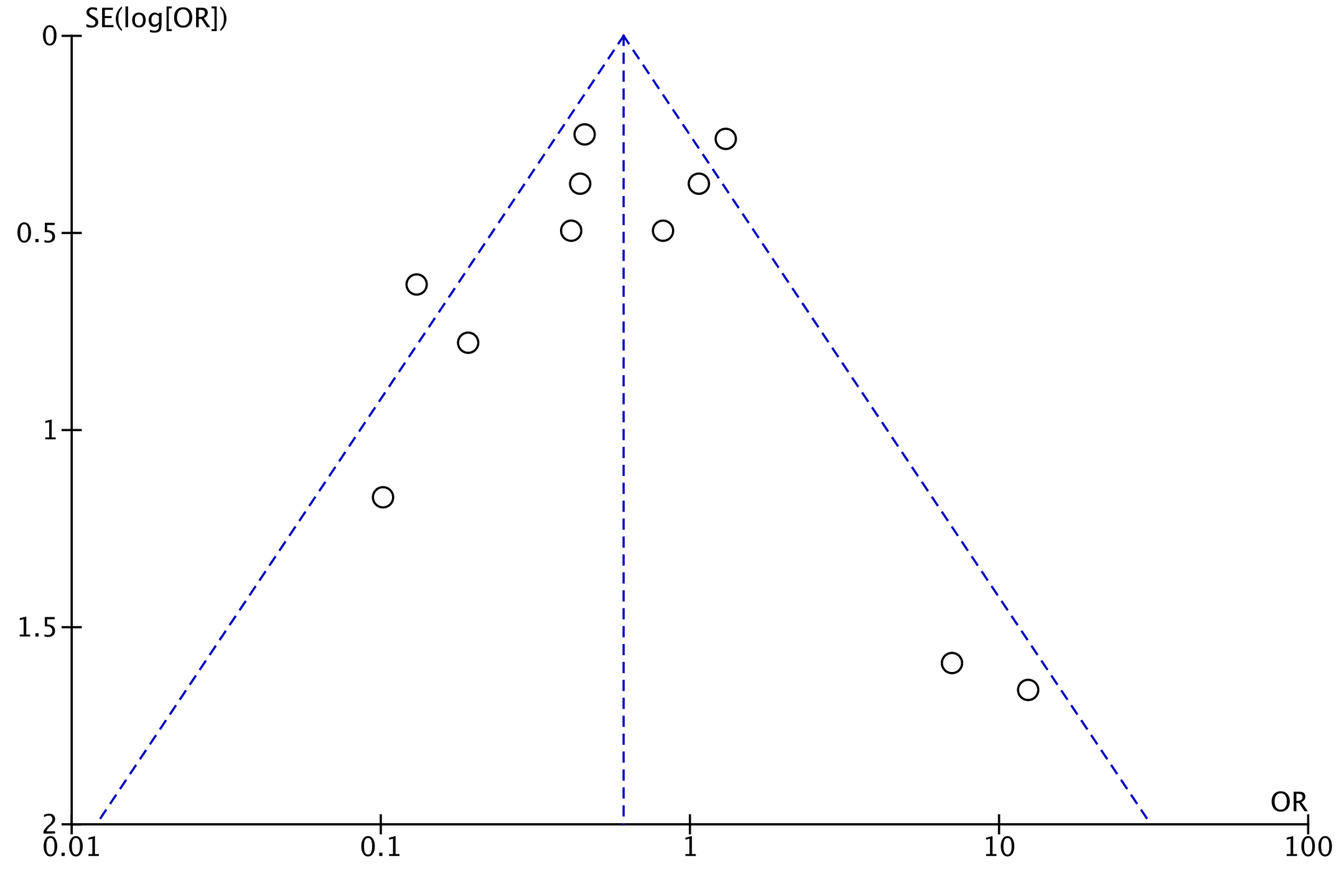


**Supplementary Figure 4(C).** Funnel plot of studies including lacunar infarcts


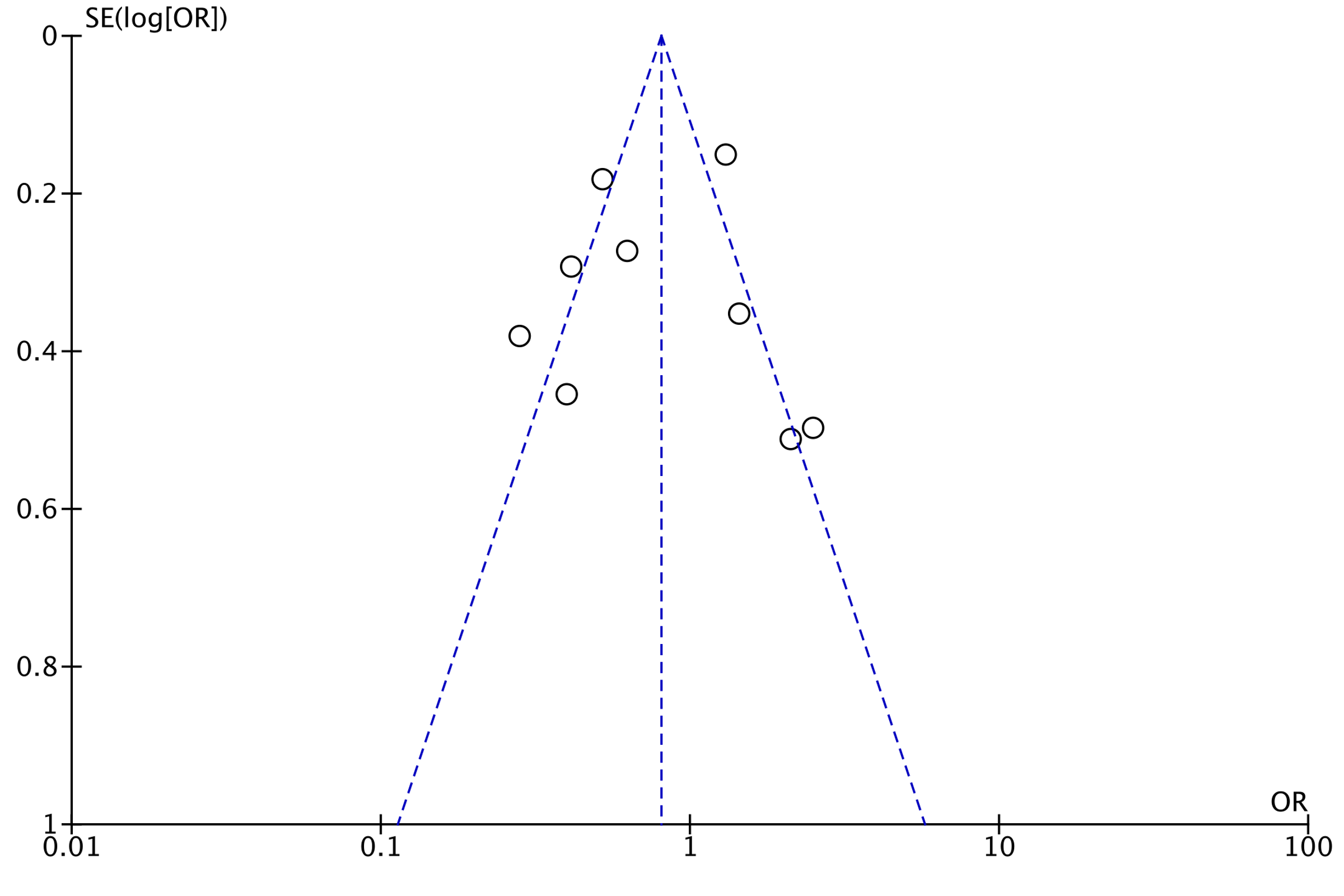


**Supplementary Figure 4(D).** Funnel plot of studies including multiple infarcts


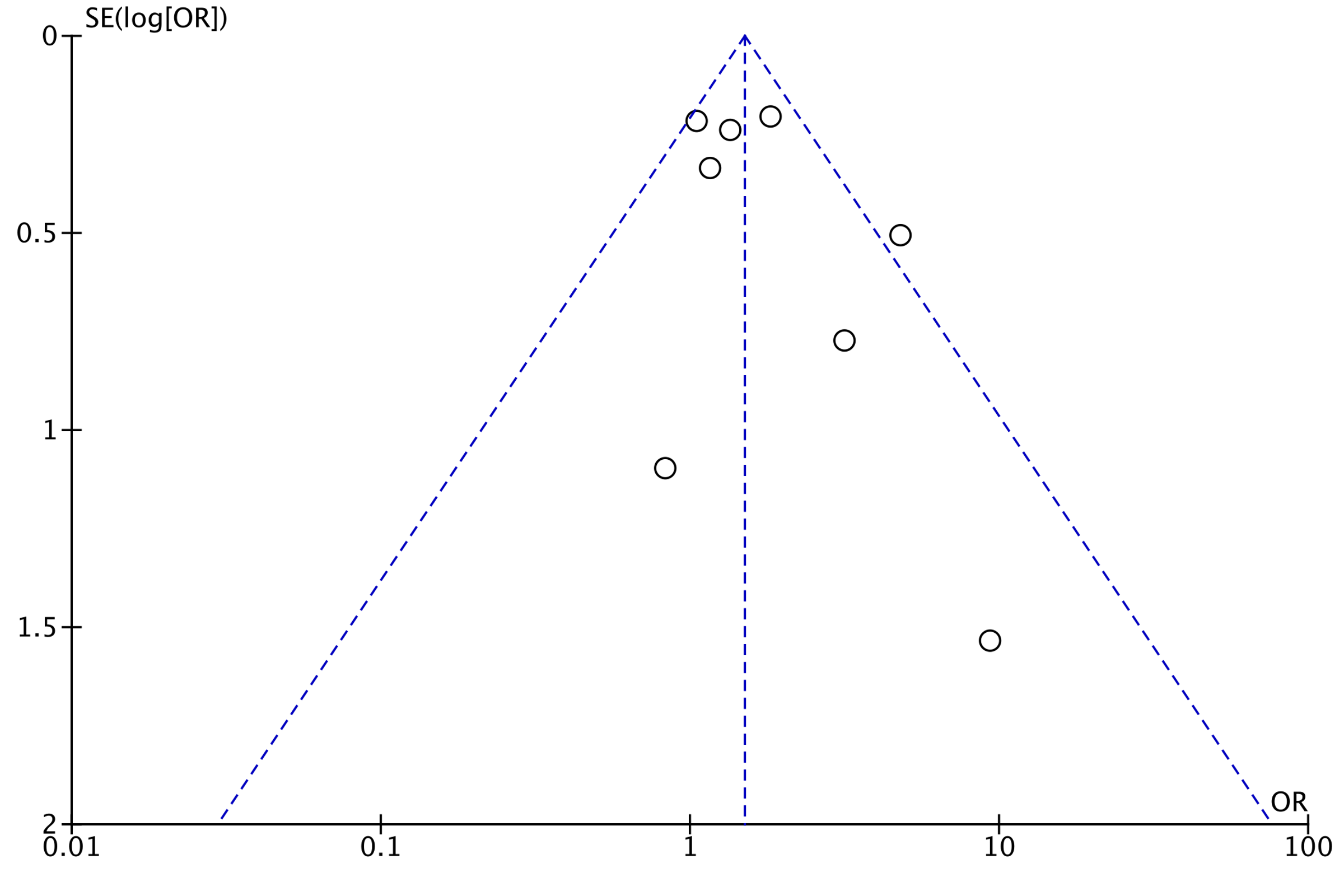


**Supplementary Figure 4(E).** Funnel plot of studies including multiple territory infarcts


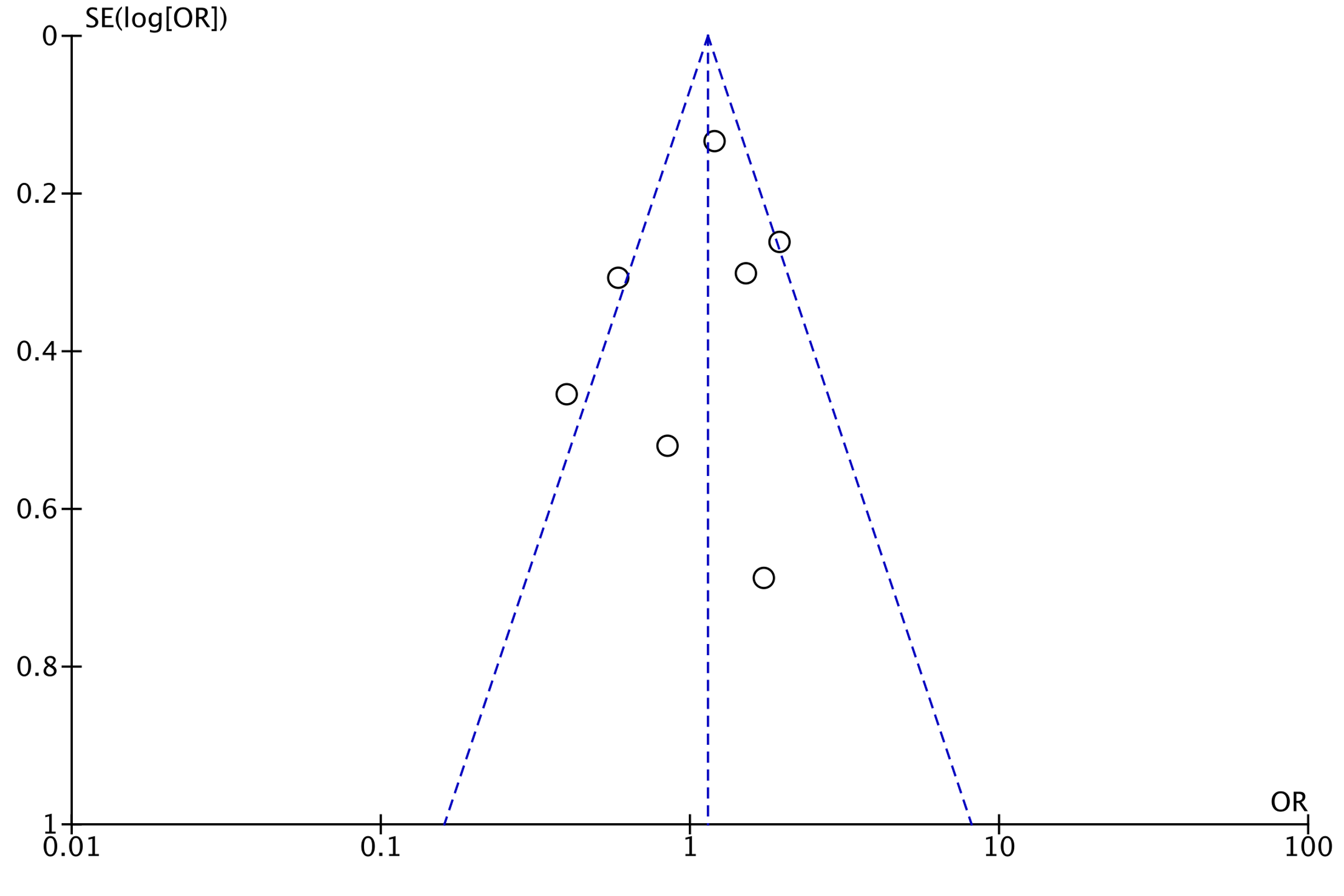


**Supplementary Figure 4(F).** Funnel plot of studies including posterior circulation infarcts
